# Effect of tobacco avoidance on the incidence of non-hereditary renal cell carcinoma in patients over 18 years of age: systematic review and meta-analysis

**DOI:** 10.1007/s11255-026-05152-x

**Published:** 2026-05-12

**Authors:** Julián Felipe Valdés Vásquez, Herney Andrés García-Perdomo

**Affiliations:** 1 UROGIV Research Group, School of Medicine, Universidad del Valle, Cali, Colombia; 2 Division of Urology/Uro-Oncology, Department of Surgery, School of Medicine, Universidad del Valle, Calle 4 B # 36-00, Cali, Colombia

**Keywords:** Smoking avoidance, Kidney neoplasms, Risk factors, Meta-analysis, Sex characteristics

## Abstract

**Objective:**

Risk factors for kidney cancer are divided into modifiable and non-modifiable factors. Among the former, smoking stands out, whose relationship with the disease has been extensively studied, from the intensity of consumption to the effects of cessation. However, the impact of avoiding smoking is not precisely known. This study aimed to estimate the effect of avoiding tobacco use on the risk of renal cell carcinoma (RCC) in patients over 18 years of age with no family history.

**Methods:**

A systematic review of clinical trials and observational studies was conducted in the Medline, Embase, CENTRAL, and LILACS databases. Patients aged 18 years or older with a confirmed histological diagnosis of RCC were included. The primary outcome was RCC development. Risk of bias was assessed using the Newcastle–Ottawa tool, and a meta-analysis of the included studies was performed.

**Results:**

Of 49 studies identified, 7 met the inclusion criteria, totaling 4,430 patients. In the total population, tobacco avoidance was associated with a lower risk of RCC (RR = 0.69; 95% CI 0.56–0.84; *I*^2^ = 20%). In the analysis by sex, men showed an RR of 0.52 (95% CI 0.32–0.85; *I*^2^ = 57%), while in women the RR was 0.83 (95% CI 0.48–1.45; *I*^2^ = 61%) and did not differ significantly.

**Conclusions:**

Smoking avoidance is associated with a substantial reduction in the risk of RCC, especially in men. Further studies with uniform definitions of avoidance are needed to assess its long-term effect.

**Supplementary Information:**

The online version contains supplementary material available at https://doi.org/10.1007/s11255-026-05152-x.

## Introducción

Kidney masses can be classified as malignant, benign, and inflammatory. Malignant tumors include renal cell carcinoma (RCC), urothelial neoplasms, sarcomas, embryonal or pediatric tumors, lymphomas, and metastases [1]. RCC is the most common solid lesion of the kidney and accounts for 90% of all malignant renal tumors. RCC subclassifications include, in order of frequency, clear cell tumor (70%), papillary (10–25%), chromophobe (5%), collecting duct, and unclassified RCC (< 1%) [2].

RCC is the sixth most diagnosed cancer in men and the tenth in women, accounting for 5% and 3% of all cancer diagnoses, respectively. Its incidence has increased in recent decades, attributable to the advent of new diagnostic imaging technologies, which, in turn, have increased its incidental detection. Approximately 17% of patients have metastatic disease at the time of diagnosis, a significant number [3]. In 2020, there were 179,368 deaths from kidney cancer, 90% of which were from RCC. These deaths occurred predominantly in men, with an overall age-standardized rate (ASR) estimated at 1.8/100,000 [4].

RCC is characterized by the loss of tumor suppressors located on the short arm of chromosome 3. Molecularly, RCC exhibits different patterns of mutations and clinical outcomes. A key feature is metabolic reprogramming. Tobacco contains at least 70 known carcinogens, including metals and metalloids. The molecular effects of smoking on RCC differ from its effects on the lungs [5]. The literature suggests that the pathogenesis of tobacco-mediated kidney cancer stems from the creation of free radicals induced by substances contained in tobacco, which cause damage to the cell’s deoxyribonucleic acid (DNA) [6]. Carcinogens, such as cadmium (Cd) and arsenic (As), act on the proximal convoluted tubule. High levels of Cd promote the accumulation of As and copper (Cu), the latter of which stimulates oxidative phosphorylation, thereby affecting tumor growth [5].

Established risk factors, include smoking, high body mass index (obesity), metabolic syndrome, and hypertension. These risk factors are considered modifiable, and reviews address the role of physical activity, smoking cessation, and obesity control as protective factors [7–9]. Tobacco is the most widely accepted environmental risk factor and is present in 20–30% of all RCC cases. All forms of tobacco have been implicated, and the risk increases with dose and duration [10]. Overall survival and progression-free survival are higher in RCC patients who quit smoking than in those who continue smoking, thus showing an apparent direct relationship [11].

Despite the clear association between smoking and renal cell carcinoma, the effect of smoking avoidance on the disease remains unknown, which could otherwise support the development of public health policies. We aimed to estimate the impact of avoiding smoking on the risk of renal cell carcinoma (RCC) in patients over 18 years of age with no family history.

## Methods

We conducted this systematic review in accordance with the recommendations of the Cochrane Collaboration and following the Preferred Reporting Items for Systematic Reviews and Meta-analyses (PRISMA) statement. The review protocol was registered in the International Prospective Register of Systematic Reviews (PROSPERO): CRD420261319710.

### Eligibility criteria

We included clinical trials, quasi-experimental studies, cohort studies, and case–control studies that enrolled patients aged 18 or older and described the association between tobacco prevention and the development of histologically confirmed RCC, regardless of cancer subtype. There were no restrictions on setting or language. We excluded studies involving patients with hereditary RCC or with tumor confirmation other than RCC.

### Source of information

We searched Medline (OVID), Embase, LILACS, and the Cochrane Central Register of Controlled Trials (CENTRAL) from their inception to the present (Appendix 1). To ensure saturation of the literature, we reviewed references from the most relevant articles identified through our research, conferences, thesis databases, Open Grey, Google Scholar, and ClinicalTrials.gov, among others. Authors were contacted by email to check whether any information was missing.

### Data collection

Two researchers reviewed each reference by title and abstract, then examined the full texts of relevant studies, applied pre-specified inclusion and exclusion criteria, and extracted data. Disagreements about the inclusion of articles were discussed and resolved. When it was not possible to fix them, agreements were reached to resolve the conflict.

Two trained reviewers, using a standardized form, independently extracted the following information from each article: title, year of study, authors’ names, location, design, objectives, population, inclusion and exclusion criteria, methodology, blinding, follow-up time, interventions and comparator, outcomes, losses to follow-up, results, and measures of association.

### Risk of bias assessment

Given the nature of the included studies, the risk of bias was assessed using the Newcastle–Ottawa Scale. Two independent researchers evaluated the potential risk of bias based on the information extracted, rating it as “good quality,” “medium quality,” or “poor quality.”

### Statistical analysis

We performed the statistical analysis in Review Manager 5.3 (RevMan® 5.3). For categorical outcomes, risk ratios (RR) with corresponding 95% confidence intervals (95% CI) were reported. The effect size was standardized as a risk ratio comparing never smokers versus current smokers (or equivalent). When studies reported the inverse measure (e.g., odds ratio of smokers versus never smokers), the estimate was inverted (OR⁻^1^), and confidence intervals were appropriately transformed on a logarithmic scale. In addition, when both crude and multivariable-adjusted effect estimates were available, crude estimates were preferentially extracted to ensure methodological consistency across studies. Adjustment strategies varied substantially among the included studies, with heterogeneous covariate selection (e.g., age, hypertension, and body mass index were not uniformly included across models).

We pooled data using a random-effects meta-analysis, consistent with the expected heterogeneity. We presented the results in forest plots showing pooled effect estimates with 95% CIs. Heterogeneity was assessed using the *I*^2^ statistic. For interpretation, *I*^2^ values above and below 50% were considered indicative of high and low heterogeneity, respectively.

Analyses were stratified by study design (cohort versus case–control) and by sex when data allowed.

## Results

**Fig. 1:**
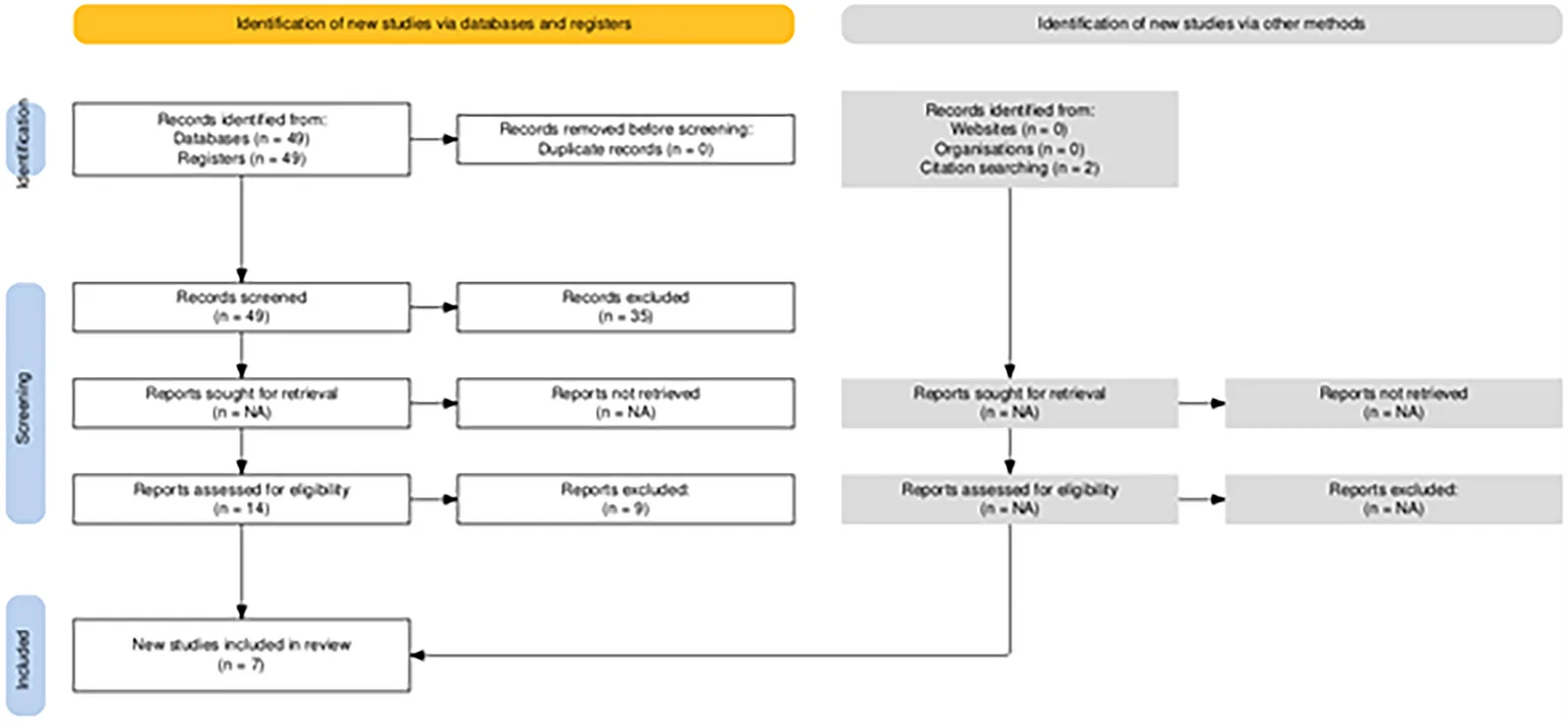
Study selection diagram

Forty-nine records were identified using search strategies, and no duplicate documents were found. The inclusion and exclusion criteria were applied, and seven articles were ultimately included for qualitative and quantitative analysis (Fig. 1).

### Characteristics of the included studies

Three case–control studies, two prospective cohorts—one based on a set of seven Australian cohorts—and two retrospective cohorts were included (see Table 1). The total number of patients with RCC was 4430. All studies evaluated the association between smoking and RCC; six of them had the diagnosis of this pathology as their primary outcome, and one study reviewed its relationship with stage, emphasizing advanced disease. McCredie et al. included patients with high urothelial cancer, a population that was not considered, and only Patel et al. (2015), described measures of association by RCC subtype. The follow-up time in the retrospective cohorts was 12 years for men and 24 years for women in the study by Flaherty et al. (2005), and 8–22 years in the study by Laaksonen et al(2020). Cote et al. (2018) was the only study to take racial factors into account when establishing measures of association. However, three of the included studies recorded this type of variable

**Table 1:** Characteristics of the included studies

Ref ID	Author	Type of study	Cases (*n*)	Controls (*n*)	Sociodemografic caracteristic								Comments
					Gender	Age	Race	BMI	Status of tobacco consumption	CCR subtype	Comorbilities	Association measures with 95% CI	
1	McCredie et al. [15]	Case and controls	489	523	Cases: 310 men and 179 women Controls: 231 men, 292 women	20–79 years old	–	Covariate	Non-smoker (< 100 cg), former smoker, current smoker Pack/year (low, medium, high) Age of onset (< or > 18 years) Years of cessation 20 cigarettes per pack	–	Obesity	Reference: non-smokers: RR: 1 Current smokers vs. non-smokers: RR = 2.17 (95% CI 1.55–3.02) Men: Current smokers: RR = 2.94 (95% CI 1.80–4.78) Women: Current smokers: RR = 1.60 (95% CI 1.00–2.56)	
2	Parker et al. [16]	Case and controls	387	2333	70% male smokers, 30% female smokers 43.1% male never smokers, 56.9% female never smokers	Age in controls: mean 64.0–72.5 years depending on group Age in cases: range 40–85 years (according to inclusion criteria)	NA	Categorized < 24, 24–29, > 29	Current, former smoker, years since quitting, and never smoker Never smoker: an individual who has never used a tobacco product for 6 months or more	–	Hypertension, high BMI	Current smokers: OR 1.0 Never smokers: OR = 0.6 (95% CI 0.4–0.9)	
3	Flaherty et al. [17]	Prospective cohort	266	–	156 women and 110 men	Average age: women 42.4 years (30–55 years), men 54.0 years (40–75 years)	NA	BMI at baseline and updated; weight at age 18 (women) or 21 (men) 8.1% of women BMI > 30 7.8% of men BMI > 30	Never smoked, former smoker, and current smoker—pack a year	Clear cells (60%), papillary, chromophobe, collecting duct 12%, and unclassified (28%)	Hypertension, obesity Women: 11.4% HTN, 23.2% former smokers, 33.1% current smokers Men: 22.2% HTN, 41.8% former smokers, 9.6% current smokers	Reference: non-smokers: RR: 1 Men: Current smokers: RR = 1.3 (95% CI 0.6–2.9) Women: Current smokers: RR = 0.9 (95% CI 0.6–1.5)	Tables 1 and 3 show associations for BMI and hypertension
4	Tsivian, [22]	Prospective cohort	845	–	540 men	Median age 60 years for non-smokers, 65 years for former smokers, 56 years for current smokers	204 African Americans	No report	51.5% never smokers, 29.1% former smokers, 19.4% current smokers Status, duration, intensity, cumulative exposure, and cessation	CCR (clear cell 77%, papillary 19%, other 4%) Metastatic, > T3 and/or lymph node involvement	Personal and family history of cancer	Smoking status (compared to never smokers): Current smokers: OR = 1.59 (p = 0.033)	
5	Cote et al. [18]	Case and controls	1217	1235	Cases: 720 men 497 women Controls: 689 men 546 women	20–79 years old	Cases: 361 African Americans and 856 Caucasians Controls: 523 African Americans and 712 Caucasians	< 25, 25–30, 30–35, > 35 kg/m^2^	Never smoked, current smoker; pack-years; years since quitting Never smokers: less than 100 cigarettes in their lifetime Occasional smokers: more than 100 cigarettes in their lifetime but never at least 1 cigarette per day for 6 months or more More than 100 cigarettes: Former smokers or current smokers, heavy smokers if they had a pack-year > 20	Cases: 73.4% clear cell, 9.8% papillary, 11% other Controls: 57.6% clear cell, 19.6% papillary, 12.6% other	Hypertension Obesity	Reference: never smokers Smoking (Caucasians): Current smokers vs. never smokers: OR = 1.46 (95% CI 1.05–2.04), Smoking (African Americans): Current smokers vs. never smokers: OR = 1.16 (95% CI 0.81–1.65),	Modification of effect: Tables by race and HTN Obese: no significant association found; there is a table for BMI less than and greater than 30
6	Patel et al. [20]	Prospective cohort	705	–	496 men 320 women	Average among never smokers: 60.73 years Active smokers: 55.01 years Ex-smokers: 64.46 years	732 white 60 black 14: other	402 BMI > 30	Active smoker (21%), former smoker (29.8%), never smoked (49.3%)	Subtypes: ccRCC (75%), pRCC (15%), chRCC (5%) 524 clear cell 95 papillary 35 chromophobe	–	Active smoking ccRCC: OR = 2.22 (95% CI: 1.16–4.26), *p* = 0.016 pRCC: OR = 2.43 (95% CI 1.07–5.53), *p* = 0.034 chRCC: OR = 0.406 (95% CI 0.077–2.149), *p* = 0.289 Other RCC: OR = 0.39 (95% CI 0.04–3.70), *p* = 0.415	
7	Laaksonen et al. [21]	Prospective cohort of 7 cohorts	521	–	59% women	18–100 years old Average: 59 years old	NA	< 25, 25–29.9, > 30	56% never smokers, 34% former smokers, 9% current smokers	–	IMC, blood pressure, alcohol consumption included as adjustment variables	Never smokers: HR: 1 reference Active smokers: HR = 1.41 (95% CI 1.02–1.96)	Advanced age HR 1.58 (1.46–1.71) Male gender HR 1.80 (1.38–2.34) Heavier weight HR 1.10 (1.03–1.17), BMI > 30 HR 1.83 (1.44–2.33) High blood pressure HR 1.71 (1.11–2.61)

The definition of non-smokers varied between studies. Some articles did not define the categories; in others, the definition of “never smoker” or “avoidant” was a patient who had smoked less than 100 cigarettes in their lifetime or who had not smoked for more than six months.

### Characteristics of excluded studies

Studies were excluded primarily because they did not include measures of association, combine other types of genitourinary tumors, or did not address the effect of smoking avoidance.

### Assessment of risk of bias and quality of evidence

Using the Newcastle–Ottawa scale, most included studies received medium- and high-quality scores. Variability was observed in the adjustment for important confounders, such as high blood pressure and body mass index, and, in some studies, in the lack of blinding in exposure data collection. A risk of selection bias was considered in the study by Patel et al. because the population included—patients referred for surgery—may not be representative of the general population (Table 2).

**Table 2:** Bias analysis

Study		Selection				Comparability	Exposure result			
		Representativeness of cases (or exposed)	Selection of controls (or unexposed)	Confirmation of exposure (or diagnosis)	Absence of outcome (in cohorts) at the start of follow-up	Control of confounding factors	Objective or independent outcome assessment/Objective or blinded exposure validation	Sufficient follow-up time for the event to occur/Same measurement method in cases and controls	Loss to follow-up < 20% (adequately described)/No adequate response or low loss rate	TOTAL
1	McCredie et al. [15]	★	★	★	★	★★	★	★	–	8
2	Parker et al. [16]	★	★	★	★	★★	–	★	–	7
3	Flaherty et al. [17]	–	★	★	★	★★	★	★	★	8
4	Theis et al. [18]	★	★	★	–	★★	–	★	–	6
5	Tsivian, [22]	–	★	–	–	★★	★	–	★	5
6	Cote et al. [19]	★	★	★		★★	–	★	–	6
7	Patel et al. [20]	★	★	★	★	★★	★	★	★	9
8	Laaksonen et al. [21]	–	–	–	–	–	–	–	–	–

### Primary outcome

**Fig. 2:**
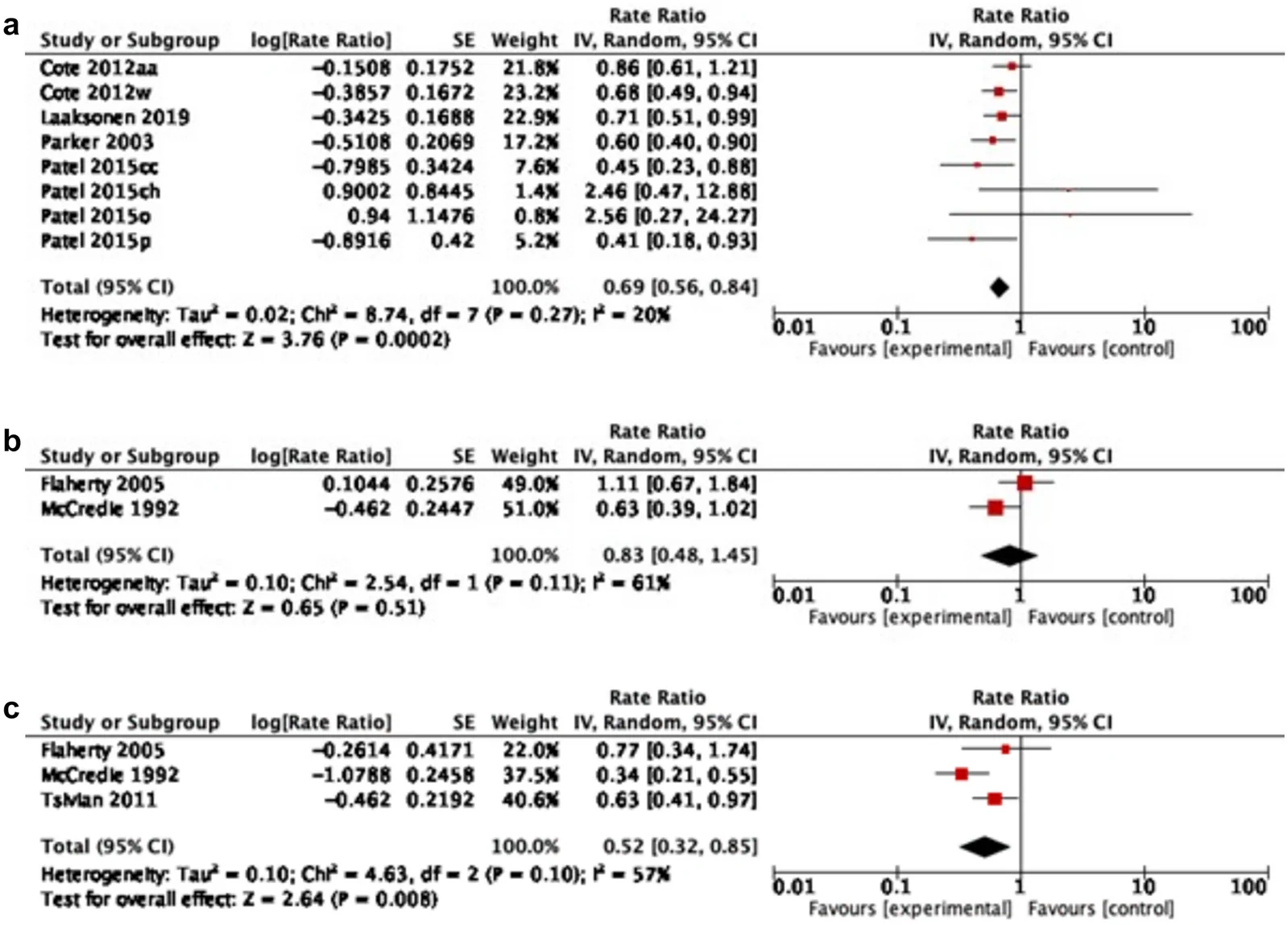
2a. Risk of CRC. Total population 2b. Risk of CRC. Women 2c. Risk of CRC. Men

Seven studies met the inclusion criteria for the review, and all provided extractable data that could be combined to compare tobacco avoidance (avoiders) vs. smokers. In the primary meta-analysis, where avoidance was defined as the category reported by each study representing the absence of consumption compared to active smokers, an RR = 0.69 (95% CI 0.56–0.84; I2: 20%) was found, showing that tobacco avoidance was associated with a lower risk of RCC (Fig. 2a).

In the analysis stratified by sex, the male group showed an RR of 0.52 (95% CI 0.32–0.85; I2: 57%), indicating a lower risk of RCC in this population, with statistically significant values. In women, the RR value of 0.83 (95% CI 0.48–1.45; I2 61%) showed no difference between tobacco.

Regarding the other variables considered, one of the studies (Laaksonen) previously described known risk factors, as advanced age and male sex were associated with HR = 1.58 (95% CI 1.46–1.71) and HR = 1.80 (95% CI 1.38–2.34), respectively. Obesity and hypertension were described by three of the included studies; only one of them found no association between obesity and the risk of RCC, while hypertension remained a constant risk factor. Cote et al. (2012) showed that smoking in white patients was associated with RCC with an OR = 1.46 (95% CI 1.05–2.04), but not in African American patients who were also smokers (Fig. 2b and c).

## Discussion

In this systematic review and meta-analysis, avoiding tobacco use was associated with a significant reduction in the risk of RCC. In the pooled analysis, individuals who had never smoked had a lower risk compared to active smokers. These findings are consistent with the well-established role of tobacco exposure as a modifiable risk factor for RCC and suggest that smoking avoidance may be associated with a reduced incidence of this malignancy, highlighting the importance of prevention and cessation strategies as public health measures with a potential impact on the incidence of this cancer. Parker and Tsivian et al. showed a decrease in RCC risk proportional to the time since cessation. The findings were consistent with a preventive effect of tobacco avoidance on RCC incidence; however, heterogeneity by sex and methodological limitations (heterogeneous definition of “avoidance,” possible residual confounding, dependencies between subgroups within the same study) should be considered when interpreting the evidence.

It should be noted that some studies contributed subentries by histological subtype that yielded imprecise estimates (wide confidence intervals) and made little contribution to the total weight of the meta-analysis. A sensitivity analysis was performed excluding these subentries (not independent), and the overall protective effect remained, although the magnitude varied slightly.

When stratified by sex, the magnitude of protection was greater in men. At the same time, in women, the association did not reach statistical significance. This difference may be due to variations in cumulative exposure intensity, biological differences in carcinogen metabolism, or lower statistical power in studies that included female populations. Further research with greater representation of women is needed to elucidate whether there are actual differences in biological susceptibility or whether methodological factors explain the variability.

The findings are consistent with previous studies documenting the association between tobacco and RCC and its dose-dependent nature. One of these studies, the Vital and Lifestyle Study (VITAL), which included 77,260 patients, revealed that a smoking index of 22.5 packs/year represented more than a 50% risk for RCC compared to non-smokers [12]. Similarly, the Prostate, Lung, Colorectal, and Ovarian (PLCO) trial, which involved 148,000 men and women aged 55 to 74, showed that smoking was significantly associated with an increased risk of developing RCC and a higher grade [13].

In 2015, Cumberbatch et al. found that the pooled RR for RCC incidence was 1.31 (95% CI: 1.22–1.40) for all smokers, 1.36 (95% CI 1.19–1.56) for current smokers, and 1.16 (95% CI 1.08–1.25) for former smokers. The disease-specific mortality (DSM) risk was 1.23 (95% CI: 1.08–1.40), 1.37 (95% CI 1.19–1.59), and 1.02 (95% CI 0.90–1.15), respectively [6]. Addressing dose dependence, a systematic review of 56 studies conducted by Liu et al. found a nonlinear increased risk with smoking intensity; the RR compared to never smokers was 1.18 (95% CI 1.11–1.26) for five and 1.72 (95% CI 1.52–1.95) for thirty cigarettes/day [10].

Sex-specific differences in the association between tobacco exposure and RCC risk likely reflect a convergence of biological and epidemiological factors. Metabolically, tobacco-derived carcinogens undergo renal bioactivation via cytochrome P450 enzymes (CYP1A1, CYP1A2, CYP2E1), generating reactive oxygen species and DNA adducts that initiate carcinogenesis. Sex-based differences in constitutive CYP450 activity may result in differential carcinogen load at the renal tubular level. Additionally, null genotypes for glutathione S-transferase isoforms GSTM1 and GSTT1 appear more frequently in male-enriched populations, potentially amplifying tobacco-related renal damage. Estrogens may further attenuate carcinogenesis through estrogen receptor-mediated suppression of HIF-1α signaling, a pathway central to clear cell RCC, and through reduction of oxidative stress and angiogenic activity [6, 14].

From an epidemiological standpoint, men in the included studies generally exhibited higher cumulative tobacco exposure, earlier smoking initiation, and more frequent occupational exposure to nephrotoxic agents, such as trichloroethylene and cadmium. Residual confounding by hypertension and obesity, both differentially distributed by sex and inconsistently adjusted across studies, may further distort sex-stratified estimates. These patterns are consistent with findings from the VITAL study and the Prostate, Lung, Colorectal, and Ovarian (PLCO) Cancer Screening Trial, both of which reported stronger smoking-associated RCC risk in men, with associations in women remaining directionally consistent, but not statistically significant [12, 13]. Collectively, the observed sex differential likely reflects a genuine biological gradient compounded by inequitable exposure distributions and heterogeneous confounder adjustment across studies [6, 14].

Our results should be interpreted with several limitations in mind. First, the definition of “avoidance” was inconsistent. Some studies distinguished between never smokers and former smokers, while others did not provide a clear definition of these categories. The category of never smokers may, in fact, have been that of “former smokers.” This phenomenon could attenuate the protective effect, given that ex-smokers maintain a residual risk depending on the time since cessation. Second, pooled estimates were based on crude measures of association. Although several included studies reported multivariable-adjusted analyses, adjustment strategies were inconsistent and heterogeneous across studies. Some studies adjusted for hypertension, others for body mass index or age, and only a minority incorporated multiple established RCC risk factors simultaneously. Due to this variability, pooling adjusted estimates was considered methodologically inappropriate. Consequently, residual confounding by established risk factors, such as age, obesity, and hypertension, cannot be excluded. Given the observational design of the included studies, causal inference should be avoided, and the results should be interpreted as associations rather than proof of a protective effect. Third, the relatively limited number of studies reduced the ability to explore sources of heterogeneity using other techniques and to assess publication bias robustly.

## Conclusion

Smoking avoidance was associated with a lower risk of RCC, particularly in men; however, given the observational nature of the included studies, these findings should be interpreted as associative rather than causal. These findings support the implementation of tobacco prevention as part of renal cancer control strategies and underscore the need for future prospective studies that use uniform definitions of exposure, include sufficient female representation, and explore in detail the effects of long-term avoidance and differences by histological subtype.

## Supplementary Information

Below is the link to the electronic supplementary material.

Supplementary file1 (PDF 174 KB)

## Data Availability

No datasets were generated or analysed during the current study.
