# Supplementary material for: Effect of tobacco avoidance on the incidence of non-hereditary renal cell carcinoma in patients over 18 years of age: systematic review and meta-analysis
Source: Int Urol Nephrol. 2026 May 12;58(10):4027–38. doi: 10.1007/s11255-026-05152-x (PMC13585962; doi:10.1007/s11255-026-05152-x)

PRISMA 2020 flow diagram for new systematic reviews which included searches of databases, registers and other sources

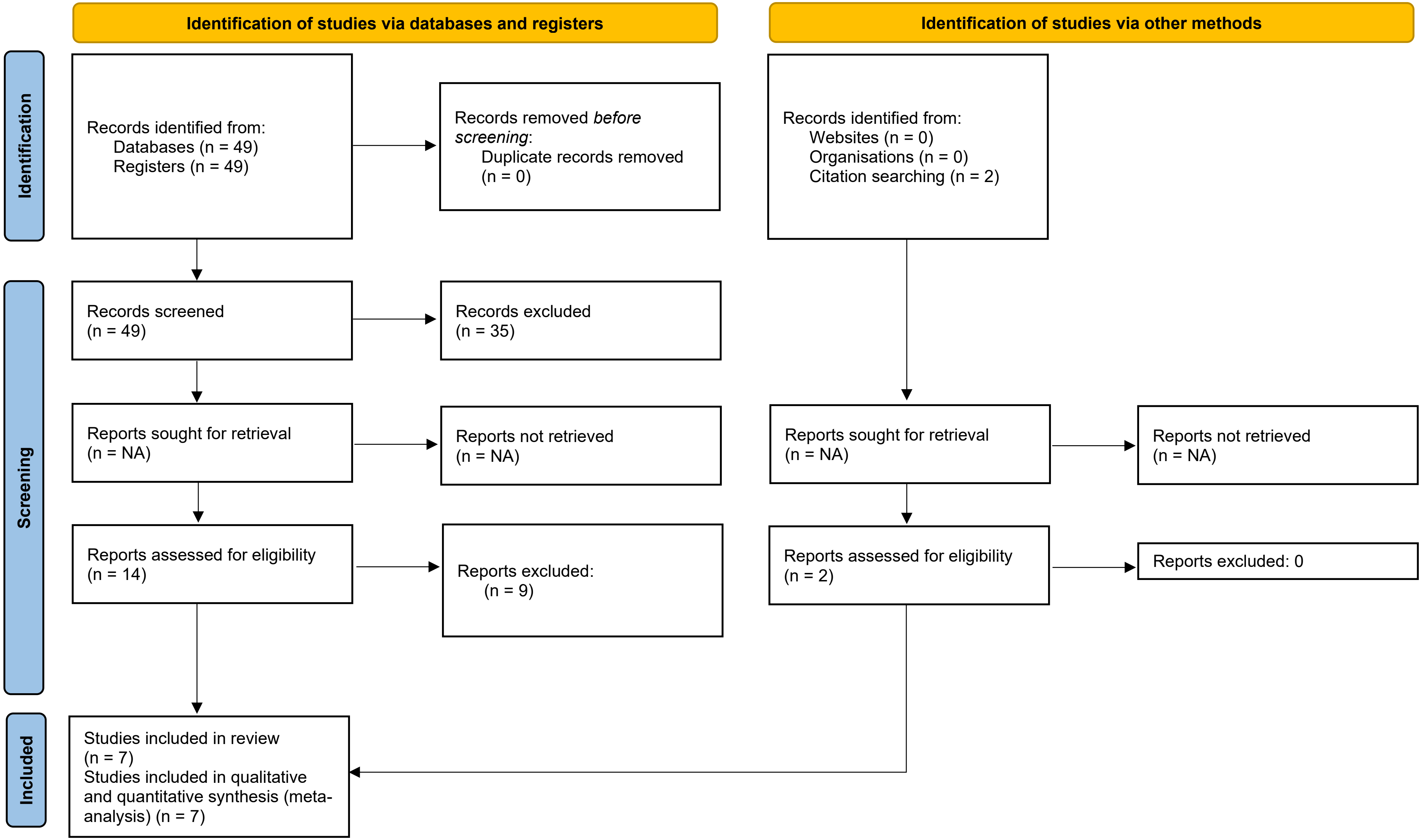

Supplement: Supplementary file 1 [file 11255_2026_5152_MOESM1_ESM.pdf]
